# Parents’ tacit knowledge of their child with profound intellectual and multiple disabilities: A qualitative study

**DOI:** 10.3109/13668250.2024.2336084

**Published:** 2024-04-23

**Authors:** Kasper Kruithof, Maartje Hoogsteyns, Ilse Zaal-Schuller, Sylvia Huisman, Dick Willems, Appolonia Nieuwenhuijse

**Affiliations:** a Department of Ethics, Law & Humanities, Amsterdam UMC, University of Amsterdam, Amsterdam, the Netherlands; b Department of Paediatrics, Amsterdam UMC, Amsterdam, the Netherlands; c Prinsenstichting, Institution and Outpatient Clinics for People with Intellectual Disabilities, Purmerend, the Netherlands

**Keywords:** profound intellectual and multiple disabilities, tacit knowledge, experiential knowledge, parents, caregivers, care

## Abstract

**Background:**

Parents’ tacit knowledge plays an important role in the care of persons with profound intellectual and multiple disabilities (PIMD). As little is known about its nature and use, we aimed to explore this parental tacit knowledge.

**Method:**

We conducted semi-structured interviews with parents (*n* = 11) about their tacit knowledge of their child, based upon video recordings they made of their child’s behaviour, and analysed the data thematically.

**Results:**

Parents described their tacit knowledge as the capacity to read their child’s subtle signs, or to sense and “just know” their child’s situation. They had developed this knowledge because of their shared history of proximity and interaction and emphasised that it was crucial in ensuring their children’s needs are met.

**Conclusions:**

We describe how parents’ tacit knowledge contributes to “good care” for persons with PIMD, interpret the implications for (medical) care practice, and discuss ways to deal with its limitations.

## Introduction

Persons with profound intellectual and multiple disabilities (PIMD) have little or no understanding of verbal language, and no apparent symbolic interaction with objects (Nakken & Vlaskamp, 2007). Their communication is mostly presymbolic or protosymbolic, such as movements, sounds, body postures, facial expressions or muscle tensions (Maes et al., 2007). This means, among other things, that proxies have a pivotal role in understanding the communication, preferences, needs and wellbeing of persons with PIMD (Nieuwenhuijse et al., 2023). While physiological measures could be helpful to clarify the situation of persons with PIMD (Vos et al., 2010), there is always interpretation needed of what they want, need, or feel (Nieuwenhuijse et al., 2023; Phelvin, 2013).

Parents fulfil an important role in interpreting their child’s situation, and consequently in defending their interests. Parents have reported before that they, as experts of their child, can “sense” that their child is not doing well, that they can “read” their child, or that they “just know” how their child feels (Carter et al., 2017; Kruithof et al., 2020; Olsman et al., 2021; Zaal-Schuller et al., 2016). We have, in line with Reinders (2010), and based on the work of Polanyi (2009) described this sensing, reading, and just knowing as tacit knowledge (Hoogsteyns et al., 2023; Kruithof et al., 2020). Such tacit knowing is a form of implicit and personal, sometimes embodied, knowing, which is difficult to explicate or share with others (Polanyi, 2009). In relation to the care of persons with PIMD, this tacit knowledge is built during intimate and repeated interactions between the caregiver and the persons with PIMD (Hoogsteyns et al., 2023).

While the implicitness of tacit knowledge may complicate the way it is valued by others, it does seem to play an important role in the care and support of persons with PIMD (Hoogsteyns et al., 2023; Kruithof et al., 2020). Parents may use their tacit knowledge to read the subtle signs of their child and estimate what their child means, wants, needs, or feels (Hostyn & Maes, 2013; Kruithof et al., 2021; Phelvin, 2013). In making such estimations, parents, for example, aim to optimise care routines for their child and consequently increase the quality of life (QoL) of their child (Hoogsteyns et al., 2023). Moreover, parents may use their tacit knowledge to assess their child’s wellbeing, or lack thereof, and their (medical) interests (Kruithof et al., 2022; Zaal-Schuller et al., 2016; Zaal-Schuller et al., submitted). Regardless of its suggested importance (Hoogsteyns et al., 2023; Reinders, 2010), tacit knowledge remains a difficult phenomenon, and may not be readily accepted as valid knowledge by others, as it is difficult to put in words (André et al., 2002; Kruithof et al., 2020; Polanyi, 2009). This may complicate communication between parents and (medical) professionals because parents feel they have to translate their tacit forms of knowledge into more explicit and objective terms to be accepted as valid knowledge by professionals (Carter et al., 2017).

In sum, parents’ tacit knowledge plays an important role in the care and support of persons with PIMD, but simultaneously is a fragile type of knowledge as it is difficult to put in words or share with others. Therefore, with the aim of improving care and support of persons with PIMD, in this study, we explored how parents use, build, and share tacit knowledge with others. We did this by answering the following questions: How do parents describe their tacit knowledge of their child with PIMD? How do they use and build this knowledge? How do they share this knowledge with professional caregivers?

## Method

### Study design

We held semi-structured interviews with parents about their tacit knowledge related to caring for their child with PIMD. In our invitation letter, we briefly introduced the concept of tacit knowledge, which we described as an implicit form of knowing that is difficult to put into words, and invited parents for an interview about this form of knowing in relation to their child. In order to address their tacit knowledge, we asked parents, before the interview took place, to video record subtle nuances in behaviour and reactions of their child that they expected to be potentially missed or misread by other caregivers. We expected that reflecting on such video recordings would enable parents to make their personal and implicit interpretations of their child’s situation and communication explicit (Hoogsteyns et al., 2023), which would allow us to explore the tacit dimension of their interpretations. In the semi-structured interviews that followed we asked parents about their interpretations of the recorded specific subtle nuances in behaviour and reactions of their child, and about their tacit knowledge about their child in general.

### Recruitment

Parents were recruited with the help of 2CU, an association of parents of children with PIMD in the Netherlands. They disseminated our invitation letter among their members. Additionally, we included parents through our own network, built during previous research projects. We included parents of children with PIMD (developmental age <2 years) who lived at least half of the time in the family home as we expected that these parents would have particular knowledge about their child that other caregivers would possibly lack. Moreover, we sampled purposively to include parents of children from different age groups, as we expected that the duration of the relationship between parent and child may be of influence on the tacit knowledge of parents (Kruithof et al., 2021).

### Data collection

Parents made their own video recording and sent the recording to either KK or AN or mentioned to KK or AN that they would show the recording during the interview. After this KK or AN made an interview appointment with the parent. We conducted semi-structured interviews with parents, at their homes, between May 2022 and December 2022. On one occasion, the mother and father were interviewed together. The interviews lasted a mean of 70 min (range, 50–100 min).

We used an interview guide with open-ended questions about parents’ (tacit) knowledge of their child with PIMD; the guide was based on two previously conducted literature studies, one on parents’ knowledge of their child with PIMD (Kruithof et al., 2020), and one on tacit knowledge in the care for persons with PIMD (Hoogsteyns et al., 2023). We started each interview by briefly introducing the concept of tacit knowledge, as we had done in our invitation letter. As part of the interviews, we asked parents how they interpreted their child’s behaviour/reaction on the video recording, why and how they came to these particular interpretations, and how their interpretation might differ from those of others. Furthermore, we asked parents what role tacit knowledge played in their interpretations of their child’s situation and communication, how they had built this tacit knowledge, how this tacit knowledge relates to other types of knowledge in the care for their child, and how they share this tacit knowledge with others. During the interviews, we encouraged parents to give examples of situations in which their tacit knowledge played an important role in the care and support for their child, as well as examples of when their interpretations based on tacit knowledge may have been wrong. The full interview guide is to be found online as an appendix.

### Data analysis

We transcribed the interviews verbatim and analysed them thematically (Braun & Clarke, 2006) in MaxQDA. First, KK and AN familiarised themselves with the data by reading all transcripts and highlighting text segments that were relevant in relation to the research questions, which served as deductive themes (parents’ description of their tacit knowledge about their child with PIMD, how they built such knowledge, the way they use this knowledge in the care and support for their child, and potential ways of sharing such knowledge). Subsequently, both KK and AN coded all interviews in open fashion to further explore these deductive themes, as well as to make sure they accounted for all themes relevant for parents. They compared their open codes with the deductive themes in order to confirm these deductive themes as main themes. Inductive coding resulted in subcodes of the deductive themes, that is, different ways of describing, using, building and sharing tacit knowledge.

After KK and AN found consensus on the main themes and their subcodes, KK further organised parents’ quotes according to the main themes and their subcodes. Finally, these main themes, subcodes and parents’ quotes were reviewed for coherence, overlap and completeness in our interdisciplinary research group consisting of four qualitative researchers, two intellectual disability physicians, a disability care professional / educator, and a mother of a child with PIMD. This resulted in removing the subcode “increasingly complex interaction patterns” as the attached quotations were seen as lacking in clarity, and in including the subcode “affirmation by others.”

We reached data saturation on the topics presented in this paper: we found patterns consistent with previous research, we gathered rich data on the topics discussed, and the later interviews did not result in alterations to the topics discussed in this paper (Morse, 2015).

### Research ethics

According to Dutch Law on Research Involving Human Subjects, this study did not need the approval of a Research Ethics Committee, which was confirmed by the Research Ethics Committee of Amsterdam UMC, location AMC (W21_435 # 21.484). We followed the ethical principles for medical research involving human subjects as laid down in the Declaration of Helsinki (WMA, 2013). We informed eligible participants orally and by written letter about the research project and about their rights, including the right to withdraw from the study at any moment. We provided the opportunity to ask questions and obtained written and oral consent for publication. All data were anonymised to guarantee the privacy of participants. Video-recordings were deleted after each interview.

### Respondents

Eleven parents (see Table 1) participated in our study. All participants, ten mothers and one father (*n* = 11, mean age, 50 years old; range, 41–68), had a Dutch ethnic background. They all had middle to high educational backgrounds, and one of them identified as religious. The age of their children with PIMD (*n* = 10) ranged from 5 to 30 years old (mean age, 16). None of them were able to express themselves verbally. Two of them were able to make basic choices with the support of communication software based on pictogram grids with voice output, while the others were too limited in their non-verbal or symbolic communication to make use of such support tools. All of them had medical problems – such as scoliosis, epilepsy, obstipation, chronic pain, visual and/or hearing impairments – in more or less severe forms, and received medication accordingly.

**Table 1.** Respondents.

Respondent	Age range (years)	Age range child with PIMD (years)	Developmental age range child with PIMD^a^ (months)	Video recording
Mother-1	40–50	0–10	0–6	Yes
Mother-2	40–50	20–30	12–18	Yes
Mother-3	60–70	30–40	18–24	Yes
Mother-4.1	40–50	0–10	6–12	Yes
Father-4.2	60–70			
Mother-5	40–50	0–10	6–12	Yes
Mother-6	40–50	10–20	18–24	Yes
Mother-7	40–50	0–10	6–12	Yes
Mother-8	50–60	20–30	0–6	No
Mother-9	50–60	20–30	Unreported	Yes
Mother-10	40–50	0–10	0–6	No

^a^Developmental age child as reported by parent.

## Results

We will outline the results in accordance with the four main themes. First, we will focus on how parents described their tacit knowledge about their child with PIMD. Then we will describe how parents had built this knowledge. After this, we will turn to how they used this knowledge. Finally, we will discuss parents’ ways of sharing their tacit knowledge about their child with other caregivers.

### The nature of parents’ tacit knowledge

At the beginning of the interview, parents showed the recordings of their child. Two parents had not made a video-recording. One mentioned how she felt it was impossible to make such a recording, as “you cannot record sense.” The other found it too difficult to grasp her specific reading of her child’s movements and behaviour on camera, as these were “moments of split seconds.” The other parents showed recordings of subtle movements of their child, which they interpreted as intentional or unintentional signs of their child that may be missed or misread by others. For example, moments in which their child would be perceived to be under stress by others, but the mother was convinced that her child was perfectly fine. Or vice versa, moments in which parents were sure their child had an epileptic seizure that professional caregivers did not recognise as such. A mother showed recordings of subtle nuances in her child’s facial expressions, which she explained as expressions of a range of discomfort:

Here she’s getting a little stuffy and then I know, I can see it in her mouth and – now she’s shaking her head too, but now she’s really uncomfortable with it. I can see it in how she moves her mouth. In the tension in her mouth, especially I see it. While you might also think, she’s pretty laid back or something, but now she’s not laid back. Look, and here just for a moment she thought, I’m going to give you a kiss. But I can see from her mouth that she, she’s just really not comfortable. (Mother-5)

The “reading” or “sensing” of subtle signs was a prominent theme during the rest of the interviews as well:

I’m just looking at her, whether she breaths faster or shallower or – So I’m very much paying attention to breathing, I notice now, as I say that to you. But I also listen to her. So, it’s really using my senses. So, I listen to her sounds and each sound has a different kind of meaning. (Mother-1)

A mother explained how small nuances in the way her daughter laughs may mean different things, and that it is difficult for other caregivers to differentiate between those meanings:

She also has a kind of weird smile. And then almost everyone thinks she likes something. And then I hear her laugh, I saw it again on Friday. Then she’s put on her side and she has a little bit of trouble with her shoulder and I say, “Now pay attention, because she’s in pain now.” Everyone is looking at me like, “She’s smiling, isn’t she?” But that’s a different kind of laugh. To differentiate between those laughs, I think you should know her really well. (Mother-6)

Some parents reported how they just sensed or felt what their child’s situation was, and could not relate this to picking up specific signs. They “just knew,” and found it difficult to explain this knowing:

I think that’s it: feeling it or something. I find it so hard to put into words, I can hardly put it into words really. It’s a feeling. That’s why I can hardly explain it. (Mother-6)

A mother related her knowledge of her child’s situation to their symbiotic relationship. She described how she felt that her connectedness with her child made that she knew how her child felt:

Even my husband often doesn’t see it, I think. Others may see it at a later stage. Do you know what I mean? (…) Why is that? I sometimes think, I’m symbiotically connected to [name child]. I’m so entangled with her. (Mother-6)

Some parents described how “feeling” and the “reading” of signs were deeply connected with one another when they assessed the situation of their children. A mother reflected on how her capacity to “feel” the situation of her child may be actually understood as her “adding up” implicitly registered subtle nuances in her child’s situation and communication:

Somehow you feel, or see – What you do then – it’s really the very small signs that you all add up maybe I think. (Mother-7)

### Building tacit knowledge

Parents reported how they had developed knowledge of their child because of their shared history of proximity and interaction with their child. By looking at reactions of their child during interactions, they learned what worked and what did not work in the care and support for their child, and they became increasingly equipped to read their child’s subtle signs:

And the moment I see that look in her eyes, I think, that’s it. And because a lot of times the reaction came after it in kind of the same process, you start to recognise it. So I don’t think I recognised it the very first time either. But because it repeated itself very often and in certain situations, that I started to recognise that facial expression and link it, and also that it evoked something in me. A feeling like oh, that [name child], who is completely helpless there. (Mother-6)

Parents emphasised the importance of taking the time, and to be fully present to try to understand the situation, needs and communication of their child:

You need to really take the time and really look at her. Just like, who is she really? Instead of just seeing her as a little doll that can’t do anything. I think those are things that are helpful. (Mother-1)

A mother described how she gently approached her child and used touch to feel what is happening to her:

When I don’t know, then I stand with her, then I hold her and then I say nothing. And then I just hold her and then I feel what is happening and where the restlessness is. And if I think, it could be your belly, then I put my hand on her belly and then I feel her body becoming less restless, for example. (Mother-7)

A mother and a father described how you need to be able to adapt to the rhythm of their son to try to “feel” what he feels:

You have to completely eliminate yourself actually. Making yourself completely subservient to someone who can do almost nothing and does almost nothing. And so you actually have to go at the same pace with him. (Father 4.2)

And keep on feeling, because he can’t indicate anything, right. He can’t indicate anything himself. No, he’s not going to make noise, actually you have to feel what he – you have to be so empathetic that you almost feel what he feels. (Mother 4.1)

Parents emphasised “the need to want to understand” their child, while simultaneously describing how there will always be a difference in the degree of wanting this between parents and professional caregivers:

As a parent, it’s your child and you’re motivated to do that, but if you’re working somewhere, it’s very different. I think that’s also the big difference. [ …] I think that is an important point. The intention you have as a parent is different from the intention of a professional caregiver, (Mother-3)

In addition to wanting it, parents described a certain sensitivity that enabled certain professional caregivers to understand their child’s situation and needs:

There are two people that [name child] has experienced, that she also grew very attached to. And they get it […] They have a certain sensitivity. You have to be emphatic and be able to place yourself in another person’s shoes. (Mother 4.1)

Parents reported how they had become increasingly confident about the correctness of their assessments of their child’s situation and needs because they had seen how their child’s situation had developed further, which gave them insights about the correctness of their earlier gut feelings. Still, they reported that they could never be absolutely certain whether their estimations were right. A mother, however, emphasised that she did not want to linger in doubt about her interpretations of her child’s potential signs, as she saw her being confident as a precondition for being able to make interpretations at all:

And about interpreting his face, because of course people sometimes ask, ‘how do you know if he’s happy or not happy?’ […] And that can never be checked. I can never ask, “[name child], is that right?” So, if I were to start doubting that now, I’m taking away my complete foundation. So, I don’t want to start doubting that either. (Mother-8)

Another mother underscored this sentiment when she described the need to dare to go by her gut feeling, since overthinking such gut feelings merely instilled doubt in her, and these gut feelings played an essential role in swiftly reacting to changes in her son’s situation:

You really must dare to feel something. That’s what I find the hardest part. I have to – I really notice that I can’t do it just in my head. Because that, then I go, that’s where all these doubts come from. I notice what I feel first. […] You really have to dare to go by your feeling. (Mother-9)

Some parents described how affirmation of medical professionals who had trusted in their gut feelings helped them to become more confident about these gut feelings:

The nurse said: “What do you think?” And I said: “My feeling tells me that it’s really not well” And then she said, “Then that’s what it is, and you should act upon that,” And I’ve had that with physicians as well. I mean, you are her voice. (Mother-5)

### Using tacit knowledge

Parents described their capacity to recognise subtle signs of their child as a means to understand at an early stage that their child was not doing well. This enabled them to predict troublesome situations or even prevent these from materialising as they were able to act on the first signs that such a situation may occur:

But at some point, it’s too much for [name child], and then he does something with his face. He turns – It’s less than this, so he turns away a little bit and he seems to slip a little out of the capacity to make contact. But that’s what he does – He gets a certain look in his eyes – [ …] And we both know, well it’s done. He needs to get away from these stimuli and he just needs to be quietly at home. And so, we need to act now. Because if we don’t, either he’s going to be very unhappy or he’s going to get a seizure. (Father-4.2)

Parents feared that professional caregivers would miss or misread potential signs that their child was not doing well. A mother described how such difficulties in reading signs could result in suboptimal care, such as epileptic seizures being missed at the day care centre:

If you’re not paying attention. And then they look oh, yes, [name child] is sitting there, you know, and then you just don’t see it. But in the meantime, a lot is happening with him. And eventually it becomes very clear, but even then he sits still in his chair. And then when they would actually look, I think they’d see it. But then they don’t see it, they don’t see it. They just think, [name child] is sitting there and he’s pulling his face a little bit. (Mother-4)

Parents expected from professional caregivers that they would trust them as experts of their child. They emphasised that professional caregivers did not need to invent the wheel once again, as they had already done that:

And you have to take me seriously. If I explain to you as a parent, like that’s the way it is, then that is the way it is. I already have that experience, so someone else doesn’t have to do it again for another twenty-two years. (Mother-2)

Some parents described how the tacitness of their knowledge made that professional caregivers would not always accept it as valid knowledge. At the same time, parents described situations in which (medical) care professionals believed their feeling that their child was not doing well, which resulted in improved care for their child. A mother described how her gut feeling made medical specialists perform additional measurements of her daughter, which eventually showed the correctness of her gut feeling and enabled her child to receive appropriate medical care:

And that’s when a paediatrician, who I’ve never seen, who has never seen [name child], believes me, contrary to what she observes herself and still tries everything, but we don’t get anywhere. And eventually the neurologist says, we’re just going to do something of which I don’t know if it makes sense. And that’s when we found out the problem. But that’s typically one of those quests that you have to do, that you know as a parent, something isn’t right, and you just hope you get the professionals on board, because they could easily say, ma’am, you’re a poser. Then [if they are on board – KK] you can figure it out. And in this case, happily they both believed me […] and we found a solution. (Mother-2)

Some parents believed that professionals tend to medicalise the care for their child for safety reasons, while they were not always in agreement that such protocols were necessary or in the interest of their child. Several described how their tacit knowledge of their child enabled them to differentiate between situations in which medical care was necessary and when not.

Then he had 40 [degrees Celsius – KK] and then they [at the care facility – KK] immediately hung the saturation meter on him, which was slightly lower then. Heart rate higher. I think, that’s rather obvious with such a high fever. And then they have to follow a protocol, […] and they had to call a doctor. And I was like, no, I don’t want that. So, then I just went to pick him up at night, because he was staying over that night, and I thought he doesn’t need to be sent in. And the next morning there was nothing more to worry about. But I also understand that there’s a protocol. So they go by the protocol, I go by my feeling. (Mother-8)

### Sharing tacit knowledge with other caregivers

Parents described how the implicit nature of their knowledge of their child, and the small nuances in different signs of their child, made it difficult to share their knowledge with other caregivers. They mentioned how the written files about their child did not allow for their tacit knowledge to be secured:

I actually find that text is very distracting. I can’t describe it very well myself. And I also find that the nuance, which should be captured in such a file, is almost always lost in that writing. (Mother-9)

On the contrary, videos of specific situations of their child were seen as possible ways of sharing their knowledge of their child with others. Some parents described how the recording they made for this study had been an eye-opener to them, and that they would start making more recordings of their child to show to professional caregivers:

It’s also kind of an eye-opener to me. I think it’s actually not a bad idea to show a new nurse some videos, like “here you see this happening, here you see that.” I think that would indeed help and speed up their process of understanding of how [name child] reacts to things. (Mother-5)

Parents emphasised that merely showing a video was not enough to share their knowledge about their child with others. They mentioned the need to reflect on the content of the video together. A father described how he made use of videos to teach professional caregivers how to recognise the early and subtle signs of his son that point towards an upcoming seizure:

There [at the day-care facility – KK] I showed videos at one point, like “look this is happening. Yeah, do you see that eye? Do you see the eyes going up and down? Do you see what the pupils are doing?.” So, you need to point out very specifically the things of which we know he does beforehand. […] And this made that they immediately understood what we meant. (Father 4.2)

In addition to sharing their knowledge with professionals, parents described the importance of professional caregivers getting the chance to build up their own knowledge of their child, by spending time with their child, trying new things and reacting to perceived signs of their child. They mentioned the importance of allowing professional caregivers the space to learn, and to instil self-belief in them. Just as they had to learn themselves, professional caregivers needed to learn to trust their gut feeling as well:

I know from myself that my own insecurity got in the way. That may be why I focus so much on empowering self-belief in other persons. Because I have seen that this insecurity has cost me a lot of energy. I have very often thought I was crazy. I have very often thought that no one would believe me. (Mother-9)

## Discussion

### Summary of findings

In this study, we explored parents’ tacit knowledge of their child with PIMD. Parents described their tacit knowledge as their capacity to (1) understand, and differentiate between, the subtle nuances in their child’s signs, and/or to (2) sense, feel and just know what their child wants, means, needs, or is experiencing. Parents reported how they had developed knowledge of their child because of their shared history of proximity and interaction and that they had become more confident about their interpretations of their child’s situation over time. Parents emphasised the importance of taking the time, to be fully present and to adapt to the rhythm of their child to intentionally read or sense their child’s situation, needs and communication. Parents emphasised that their tacit knowledge was crucial in ensuring their child’s needs are met, and asserted that it, at times, enabled them to differentiate between situations in which medical care was necessary and when not. Parents described how the implicit nature of their knowledge of their child, and the small nuances in different signs of their child, made it difficult to share their knowledge with others. Reflecting with others about videos of specific situations of their child was seen as a possible way of sharing parts of their knowledge of their child with others.

### Strengths and limitations

We described tacit knowledge to parents as an implicit form of knowing that is difficult to put in words and asked them to video-record subtle nuances in behaviour and reactions of their child that they expected to be potentially missed or misread by others. This could be understood as a limitation of our methodology as parents’ capacity to read these subtle nuances may be related to their tacit knowledge, but it is not necessarily the same. At the same time, our approach could be understood as a strength as it allowed us to come closer to the implicit knowledge of parents. The video recordings enabled parents to give words to their interpretations of their child’s behaviour or reactions, which they would usually not think about during an interview, which was underscored by some parents who referred to the recording as an eye-opener.

Our modest sample size could be regarded as a limitation as well. However, we did reach data saturation and our previously conducted literature review on tacit knowledge in caregiving dyads (Hoogsteyns et al., 2023) allowed us to identify our findings as consistent with previous findings. The underrepresentation of fathers is another limitation of our sample. Future research could aim to specifically elicit fathers’ views on their (tacit) knowledge about their child with PIMD (Dunn et al., 2021). This may prove particularly interesting since some mothers mentioned that their symbiotic entanglement with their child made them even more sensitive for signs from their child than the father. On a related note, the homogeneous ethnic background of respondents is a limitation of this study, which necessitates future studies in migrant populations.

### Subtle signs and just knowing

Parents described their tacit knowledge about their child with PIMD in two ways. First, as their capacity to understand, and differentiate between their child’s subtle signs. Parents’ tacit knowledge can in this light be understood as their (implicit) understanding and categorisation of their child’s pre-linguistic forms of signifying, such as bodily utterances, gestures and emotional expressions (Merleau-Ponty, 2012 [1945]), which they developed through years of experience with their child. Second, parents described their tacit knowledge as their capacity to sense, feel and just know what their child wants, means, needs, or is experiencing.

Some parents mentioned, while reflecting on their knowledge, that their sensing and just knowing may be understood as an extension of their capacity to differentiate between subtle signs. In this light, the gut feeling of a parent may be understood as “adding up” implicitly registered nuances in signs related to their child’s situation and communication (Stolper et al., 2009). This would mean that even what is felt or sensed is to be seen as an analytical way of knowing, from “without through perception” (Bergson, 1908/1991), which reflects a more objectivist epistemology in which reality is solid, potentially measurable and to be categorised, although this categorising may happen implicitly.

Other parents, contrastingly, reported that their sensing and just knowing were not to be explained by an implicit integration of subtle signs, but were rooted in, and only possible because of, their deep connection with their child and their openness and willingness to sense. This meant that they reported struggling to give words to their ways of knowing their child. The way these parents described their tacit knowledge points towards a more immediate way of knowing through affection and (symbiotic) entanglement; as reaching into the heart of someone through empathy and connectedness (Bergson, 1908/1991).

### Tacit knowledge and good care practice

That parents’ tacit knowledge of their child with PIMD can be understood as either their capacity to understand, and differentiate between their child’s subtle signs, or as a more direct way of sensing their child’s situation, has consequences for the way we define “good care” for persons with PIMD. The former conceptualisation of tacit knowledge is related to expertise (Evans & Collins, 2008; Pope et al., 2003), and seems less tacit than the latter form. While the recognising of subtle nuances in signs may be enhanced through (video)-observations and reflections (Hunt et al., 2003; Phelvin, 2013), the sensing, if understood as a different form of knowing, may be truly personal knowledge. This sensing may, even more so that the recognising of subtle signs, depend on the degree of attunement (Forster & Iacono, 2014; Hostyn & Maes, 2013) that can be achieved resulting from the emotional connectedness with the individual with PIMD (Watson et al., 2017).

This implies that professional care organisations should aim to create space for observation and reflection, but also foster a culture of emotional involvement towards clients. The idea of professional distance (Green et al., 2006) would, from a tacit knowledge perspective, result in suboptimal care, as this would hamper professionals’ potential for “solicitude” and “being concerned,” which results in “indifference” and thus the inability to “disclose” the situation and needs of clients (Heidegger, 2010 [1927]). This being said, there will always be a difference between parents’ and professional caregivers’ emotional connectedness with a person with PIMD. This was emphasised by the parents in our study, who described this “wanting to understand” as a precondition to develop tacit knowledge about their child. In this sense it is not surprising that parents emphasise that they have specific and particular knowledge about their child with PIMD (Kruithof et al., 2020), which professional caregivers should take seriously (De Geeter et al., 2002; Stringer et al., 2018).

### Evaluating tacit knowledge in (medical) care

While tacit knowledge could be seen as crucial in providing “good care,” especially for persons who cannot clearly communicate (Hoogsteyns et al., 2023; Kruithof et al., 2020; Reinders, 2010), it also has its limitations or even potential dangers. The first and foremost limitation of tacit knowledge is that it is difficult to explicate or validate (Gourlay, 2006). It is partly a matter of belief, which is underscored by our respondents who mentioned trust in their tacit knowledge, both from themselves and others, as an important precondition for understanding their child. However, this simultaneously means that tacit knowledge could result in (repeating) misinterpretations of the situation of a person with PIMD. Hoogsteyns et al. (2023) warn for this potential conservative element of tacit knowledge, and Olsman et al. (2021) stress that while trust must be the basis when parents “testify” on behalf of their child, there must be room for “suspicion” of (medical) professionals as well.

Notwithstanding the potential weaknesses of tacit knowledge, parents may substantiate their interpretations with previous experiences, and their interpretations and gut feelings may, until some degree, be proven right or wrong down the line, as we have seen in our findings. Reflecting with parents about their interpretations and gut feelings could help to explicate parts of their tacit knowledge (Hoogsteyns et al., 2023; Phelvin, 2013; Schutz, 2007). In such an open climate, in which parents are invited to share and discuss their interpretations of the situation of their child with PIMD, and are encouraged to relate these interpretations to previous experiences, tacit knowledge can be strengthened and its conservative element can be diminished (Hoogsteyns et al., 2023 Phelvin, 2013; Schutz, 2007). This would ideally increase the acceptability of parental tacit knowledge by professional caregivers and result in increased coproduction between parental knowledge and professional knowledge (De Geeter et al., 2002; Stringer et al., 2018). In medical settings, this would mean that tacit knowledge is used to deepen more objectivist medical knowledge (Kruithof et al., 2020). Or vice versa, measurements could strengthen or validate parents’ tacit knowledge. Such coproduction could contribute to increasingly precise interpretations of the situation and needs of individuals with PIMD and consequently result in increasingly tailored ways of caring for them. We saw examples of this in our study, namely parents who used their tacit knowledge to signal, at an early stage, when their child needed medical attention.

## Conclusion

In conclusion, the pervasive need for interpretation of the meaning of behaviour and reactions of persons with PIMD makes that we cannot demand of parents’ tacit knowledge to be proven knowledge. On the contrary, it enables caregivers to grasp what cannot be fully proven but is nevertheless important in realising “good care” for persons with PIMD as it helps to understand their subtle signs and thus interpret their situation, needs, communication, and preferences. Therefore, parents’ tacit knowledge could be understood as a way to increase the autonomy of persons with PIMD and should be regarded as crucial in their care and support.

## References

[CIT0001] André, M., Borgquist, L., Foldevi, M., & Mölstad, S. (2002). Asking for ‘rules of thumb’: A way to discover tacit knowledge in general practice. *Family Practice*, *19*(6), 617–622. 10.1093/fampra/19.6.617 12429664

[CIT0002] Bergson, H. (1991). *Matter and memory* (N. M. Paul & W. S. Palmer, Trans. & Eds.). Zone Books. (Original work published 1908)

[CIT0003] Braun, V., & Clarke, V. (2006). Using thematic analysis in psychology. *Qualitative Research in Psychology*, *3*(2), 77–101. 10.1191/1478088706qp063oa

[CIT0004] Carter, B., Arnott, J., Simons, J., & Bray, L. (2017). Developing a sense of knowing and acquiring the skills to manage pain in children with profound cognitive impairments: Mothers’ perspectives. *Pain Research and Management*, *2017*, 2514920. 10.1155/2017/2514920 PMC5385219 28458591

[CIT0005] De Geeter, K. I., Poppes, P., & Vlaskamp, C. (2002). Parents as experts: The position of parents of children with profound multiple disabilities. *Child: Care, Health and Development*, *28*(6), 443–453. 10.1046/j.1365-2214.2002.00294.x 12568473

[CIT0006] Dunn, K., Jahoda, A., & Kinnear, D. (2021). The experience of being a father of a son or daughter with an intellectual disability: Older fathers’ perspectives. *Journal of Applied Research in Intellectual Disabilities*, *34*(1), 118–128. 10.1111/jar.12791 32794330

[CIT0007] Evans, R., & Collins, H. (2008). Expertise: From attribute to attribution and back again? In E. J. Hackett, O. Amsterdamska, M. Lynch, & J. Wajcman (Eds.), *The handbook of science and technology studies* (pp. 609–630). MIT Press.

[CIT0008] Forster, S., & Iacono, T. (2014). The nature of affect attunement used by disability support workers interacting with adults with profound intellectual and multiple disabilities. *Journal of Intellectual Disability Research*, *58*(12), 1105–1120. 10.1111/jir.12103 24266858

[CIT0009] Gourlay, S. (2006). Towards conceptual clarity for ‘tacit knowledge’: A review of empirical studies. *Knowledge Management Research & Practice*, *4*(1), 60–69. 10.1057/palgrave.kmrp.8500082

[CIT0010] Green, R., Gregory, R., & Mason, R. (2006). Professional distance and social work: Stretching the elastic? *Australian Social Work*, *59*(4), 449–461. 10.1080/03124070600986010

[CIT0011] Heidegger, M. (2010 [1927]). *Being and time*. SUNY Press.

[CIT0012] Hoogsteyns, M., Zaal-Schuller, I. H., Huisman, S. A., Nieuwenhuijse, A. M., van Etten-Jamaludin, F., Willems, D. L., & Kruithof, K. (2023). Tacit knowledge in dyads of persons with profound intellectual and multiple disabilities and their caregivers: An interpretative literature study. *Journal of Applied Research in Intellectual Disabilities*, *36*(5), 966–977. 10.1111/jar.13134 37339925

[CIT0013] Hostyn, I., & Maes, B. (2013). Interaction with a person with profound intellectual and multiple disabilities: A case study in dialogue with an experienced staff member. *Journal of Intellectual & Developmental Disability*, *38*(3), 189–204. 10.3109/13668250.2013.798400 23984881

[CIT0014] Hunt, A., Mastroyannopoulou, K., Goldman, A., & Seers, K. (2003). Not knowing – The problem of pain in children with severe neurological impairment. *International Journal of Nursing Studies*, *40*(2), 171–183. 10.1016/S0020-7489(02)00058-5 12559141

[CIT0015] Kruithof, K., Olsman, E., Nieuwenhuijse, A. M., & Willems, D. (2021). ‘I hope I’ll outlive him’: A qualitative study of parents’ concerns about being outlived by their child with profound intellectual and multiple disabilities. *Journal of Intellectual & Developmental Disability*, *47*(2), 107–117. 10.3109/13668250.2021.1920377 39818581

[CIT0016] Kruithof, K., Olsman, E., Nieuwenhuijse, A. M., & Willems, D. L. (2022). Parents’ views on medical decisions related to life and death for their ageing child with profound intellectual and multiple disabilities: A qualitative study. *Research in Developmental Disabilities*, *121*, 104154. 10.1016/j.ridd.2021.104154 34954670

[CIT0017] Kruithof, K., Willems, D., van Etten-Jamaludin, F., & Olsman, E. (2020). Parents’ knowledge of their child with profound intellectual and multiple disabilities: An interpretative synthesis. *Journal of Applied Research in Intellectual Disabilities*, *33*(6), 1141–1150. 10.1111/jar.12740 32367663 PMC7687241

[CIT0018] Maes, B., Lambrechts, G., Hostyn, I., & Petry, K. (2007). Quality-enhancing interventions for people with profound intellectual and multiple disabilities: A review of the empirical research literature. *Journal of Intellectual & Developmental Disability*, *32*(3), 163–178. 10.1080/13668250701549427 17885894

[CIT0019] Merleau-Ponty, M. (2012 [1945]). *Phenomenology of perception*. Routledge.

[CIT0020] Morse, J. M. (2015). Data were saturated. *Qualitative Health Research*, *25*(5), 587–588. 10.1177/1049732315576699 25829508

[CIT0021] Nakken, H., & Vlaskamp, C. (2007). A need for a taxonomy for profound intellectual and multiple disabilities. *Journal of Policy and Practice in Intellectual Disabilities*, *4*(2), 83–87. 10.1111/j.1741-1130.2007.00104.x

[CIT0022] Nieuwenhuijse, A., Willems, D., & Kruithof, K. (2023). Understanding quality of life of persons with profound intellectual and multiple disabilities. *Journal of Policy and Practice in Intellectual Disabilities*, *21*(1). e12473. 10.1111/jppi.12473

[CIT0023] Olsman, H. J., Nieuwenhuijse, A. M., & Willems, D. L. (2021). Witnessing quality of life of persons with profound intellectual and multiple disabilities. A practical-philosophical approach. *Health Care Analysis*, *29*(2), 144–153. 10.1007/s10728-021-00428-y 33730308

[CIT0024] Phelvin, A. (2013). Getting the message: Intuition and reflexivity in professional interpretations of non-verbal behaviours in people with profound learning disabilities. *British Journal of Learning Disabilities*, *41*(1), 31–37. 10.1111/j.1468-3156.2011.00719.x

[CIT0025] Polanyi, M. (2009). *The tacit dimension*. University of Chicago Press.

[CIT0026] Pope, C., Smith, A., Goodwin, D., & Mort, M. (2003). Passing on tacit knowledge in anaesthesia: A qualitative study. *Medical Education*, *37*(7), 650–655. 10.1046/j.1365-2923.2003.01581.x 12834424

[CIT0027] Reinders, H. (2010). The importance of tacit knowledge in practices of care. *Journal of Intellectual Disability Research*, *54*(1), 28–37. 10.1111/j.1365-2788.2009.01235.x 20586882

[CIT0028] Schutz, S. (2007). Reflection and reflective practice. *Community Practitioner*, *80*(9), 26–29. 17900024

[CIT0029] Stolper, E., van Bokhoven, M., Houben, P., Van Royen, P., van de Wiel, M., van der Weijden, T., & Jan Dinant, G. (2009). The diagnostic role of gut feelings in general practice. A focus group study of the concept and its determinants. *BMC Family Practice*, *10*(1), 1–9. 10.1186/1471-2296-14-1 19226455 PMC2649045

[CIT0030] Stringer, K., Terry, A. L., Ryan, B. L., & Pike, A. (2018). Patient-centred primary care of adults with severe and profound intellectual and developmental disabilities: Patient-caregiver-physician relationship. *Canadian Family Physician*, *64*(2), 63–69. PMC5906777 29650747

[CIT0031] Vos, P., De Cock, P., Petry, K., Van Den Noortgate, W., & Maes, B. (2010). Do you know what I feel? A first step towards a physiological measure of the subjective well-being of persons with profound intellectual and multiple disabilities. *Journal of Applied Research in Intellectual Disabilities*, *23*(4), 366–378. 10.1111/j.1468-3148.2010.00553.x

[CIT0032] Watson, J., Wilson, E., & Hagiliassis, Nss. (2017). Supporting end of life decision making: Case studies of relational closeness in supported decision making for people with severe or profound intellectual disability. *Journal of Applied Research in Intellectual Disabilities*, *30*(*6*), 1022–1034. 10.1111/jar.2017.30.issue-6 28815814

[CIT0033] World Medical Association. (2013). *Declaration of Helsinki – Ethical principles for medical research involving human subjects.* World Medical Association. Retrieved March 27, 2019, from https://www.wma.net/policies-post/wma-declaration-of-helsinki-ethical-principles-for-medical-research-involving-human-subjects/

[CIT0034] Zaal-Schuller, I. H., de Vos, M. A., Ewals, F. V., van Goudoever, J. B., & Willems, D. L. (2016). End-of-life decision-making for children with severe developmental disabilities: The parental perspective. *Research in Developmental Disabilities*, *49*, 235–246. 10.1016/j.ridd.2015.12.006 26741261

[CIT0035] Zaal-Schuller, I. H., Kruithof, K., Hoogsteyns, M., Nieuwenhuijse, A. M., Willems, D. L., & Huisman, S. A. (submitted). Tacit knowledge in medical consultations for patients with profound intellectual and multiple disabilities: An exploratory qualitative study. 10.1111/jar.13134 37339925

